# Predicting factors and clinical outcome of biochemical incomplete response in middle eastern differentiated thyroid carcinoma

**DOI:** 10.1007/s12020-024-03844-x

**Published:** 2024-05-02

**Authors:** Sandeep Kumar Parvathareddy, Abdul K. Siraj, Saeeda O. Ahmed, Padmanaban Annaiyappanaidu, Maha Al-Rasheed, Wael Al-Haqawi, Zeeshan Qadri, Saif S. Al-Sobhi, Fouad Al-Dayel, Khawla S. Al-Kuraya

**Affiliations:** 1 Human Cancer Genomic Research, Research Center, King Faisal Specialist Hospital and Research Centre, Riyadh, Saudi Arabia; 2 Department of Surgery, King Faisal Specialist Hospital and Research Centre, Riyadh, Saudi Arabia; 3 Department of Pathology, King Faisal Specialist Hospital and Research Centre, P.O. Box 3354, Riyadh, 11211 Saudi Arabia

**Keywords:** Biochemical incomplete response, Differentiated thyroid cancer, Thyroglobulin, Predictive factors, Structural disease

## Abstract

**Purpose:**

The aim of this study was evaluate biochemical incomplete response (BIR) in Middle Eastern differentiated thyroid cancer (DTC), identify factors that could predict BIR before radioactive iodine (RAI) ablation and to investigate the long-term clinical outcome of DTC patient exhibiting BIR to initial therapy.

**Methods:**

We retrospectively evaluated 1286 DTCs from Middle Eastern ethnicity who underwent total thyroidectomy and RAI therapy. Demograpic and clinico-pathological factors predicting BIR were evaluated. The outcome of these patients was analyzed using primary outcome of structural disease and disease-free survival (DFS).

**Results:**

With a median follow-up of 10 years, 266 (20.7%) patients had BIR. High pre-ablation stimulated thyroglobulin (presTg), presence of lymph node metastasis, male gender and delayed initial RAI therapy (≥3 months) after thyroidectomy were significant independent predictors of BIR. Upon evaluating long-term clinical outcomes in 266 patients with BIR, we found 36.8% of patients developed structural disease. Male sex (OR = 1.56; 95% CI = 1.05–2.30; *p* = 0.0272) and increasing Tg after initial therapy (OR = 4.25; 95% CI = 1.93–10.82; *p* = 0.0001) were independent risk factors for structural disease in patients with BIR. DFS was significantly worse if both these risk factors existed concomitantly (*p* < 0.0001).

**Conclusion:**

To achieve the fair efficacy of RAI therapy, early prediction of BIR before RAI ablation is desirable. Our finding of the clinico-pathological factors (high presTg level, LNM, delayed RAI therapy and male gender) could serve as easy and robust early predictors of BIR. In addition, DTC patients exhibiting BIR had a high risk of structural disease and hence personalized management approach would be preferable for BIR patients to ensure best clinical outcome.

## Introduction

Differentiated thyroid cancer (DTC), which includes papillary and follicular thyroid cancers, is the most common endocrine malignancy and accounts for 90% of all thyroid malignancies [1–4]. Therapy modalities of DTC mainly include surgery, radioactive iodine (RAI) therapy and thyrotropin (TSH) suppression therapy [5–7]. RAI therapy plays a crucial role in the purge of potential residual thyroid cancer and reducing risk of mortality [7, 8]. DTC, with appropriate therapy, tend to have excellent prognosis [7, 9, 10]. However, recurrence and persistence can occur several decades later [11–13]. Patients having persistent radioiodine avid lesions usually require long-term surveillance and repeated high dose of RAI [14, 15]. Therefore, it is very important to identify risk factors to predict disease persistence for DTC in order to help clinician to determine the most appropriate follow-up for these patients.

One of the risk assessment tools for persistence and recurrence is dynamic risk stratification (DRS) [7, 16, 17]. Based on an optimal response to initial therapy, DRS is composed of four response groups: excellent, indeterminate, biochemical incomplete and structural incomplete. Biochemical incomplete response (BIR) classification was defined as having persistently abnormal suppressed and/or stimulated thyroglobulin (Tg) or rising anti-Tg antibodies (TgAb) without structural evidence of disease [7]. Patients exhibiting BIR have shown to have a high risk of structural recurrent and persistent disease [18, 19].

Data about BIR in Middle Eastern DTC is limited. Therefore, we conducted this retrospective study on a large cohort of Middle Eastern DTC to determine: 1. The prevalence of BIR in this cohort, 2. Clinico-pathological parameters to predict BIR after surgery and before RAI, and 3. Long-term clinical outcomes of patient with BIR. Furthermore, we tried to investigate the relationship between the timing of initiating RAI therapy and BIR in this cohort.

## Materials and methods

### Clinical cohort

One-thousand eight-hundred and twenty-two DTC patients diagnosed between 1988 and 2018 at King Faisal Specialist Hospital and Research Center (Riyadh, Saudi Arabia) were available to be included in the study. The patients were included in the study after considering the following inclusion and exclusion criteria:

#### Inclusion criteria

1. Pathologically proven DTC post total thyroidectomy
2. Received radioactive iodine ablation post surgery
3. Adequate clinical follow-up data for at least 12 months is available

#### Exclusion criteria

1. Less than total thyroidectomy (n = 63)
2. No radioactive iodine ablation received (n = 183)
3. Patients with elevated TgAb during follow-up (n = 121)
4. Patients with persistent positive WBS (n = 118)
5. Patients with follow-up duration less than 12 months (n = 51)

After applying the inclusion and exclusion criteria, 1286 DTC patients were included for the final analysis. The Institutional Review Board of the hospital approved this study and since only retrospective patient data were used, the Research Advisory Council (RAC) provided waiver of consent under project RAC # 221 1168 and # 2110 031. The study was conducted in accordance with the Declaration of Helsinki.

### Clinico-pathological and follow-up data

Baseline clinico-pathological data were collected from case records and have been summarized in Table 1. Staging of DTC was performed using the eighth edition of American Joint Committee on Cancer (AJCC) staging system [16]. Following initial surgery, all patients had pre-ablative stimulated Tg (presTg) evaluated before receiving RAI therapy. Patients were stratified into low, intermediate and high risk based on 2015 ATA guidelines [7]. Low-risk DTC patients were followed up annually, intermediate risk patients were followed up at 6 months’ intervals and high risk patients were followed up at 3 months’ intervals. At each follow-up, neck ultrasound, thyroid function tests, thyroglobulin (Tg) levels and thyroglobulin antibodies were performed. In addition, for high risk patients, whole body scan (WBS) and/or PET CT scan were performed to identify tumor persistence/recurrence. Patients with stimulated Tg of less than 10.0 ng/ml with no clinical or imaging evidence of tumors were considered complete treatment responses. BIR was considered in patients with a raised stimulated serum Tg level (>10 ng/ml) and a negative WBS.

**Table 1:** Clinico-pathological details of the study cohort (n = 1286)

Clinico-pathological characteristics	n (%)
Age at diagnosis, years (mean ± SD)	38.9 ± 16.5
Gender	
Male	322 (25.0)
Female	964 (75.0)
Tumor laterality	
Unilateral	858 (66.7)
Bilateral	421 (32.7)
Unknown	7 (0.6)
Tumor focality	
Unifocal	638 (49.6)
Multifocal	642 (49.9)
Unknown	6 (0.5)
Extrathyroidal extension	
Present	552 (42.9)
Absent	724 (56.3)
Unknown	10 (0.8)
pN	
N0	499 (38.8)
N1	664 (51.6)
Nx	123 (9.6)
Distant metastasis at diagnosis	
Present	72 (5.6)
Absent	1214 (94.4)
Stage	
I	1087 (84.5)
II	137 (10.6)
III	14 (1.1)
IV	47 (3.7)
Unknown	1 (0.1)
*BRAF* mutation	
Present	672 (52.2)
Absent	540 (42.0)
Unknown	74 (5.8)
*TERT* mutation	
Present	150 (11.7)
Absent	1010 (78.5)
Unknown	126 (9.8)
Interval to RAI therapy	
<3 months	630 (49.0)
≥3 months	656 (51.0)
ATA risk stratification	
Low	198 (15.4)
Intermediate	450 (35.0)
High	638 (49.6)

### *BRAF* and *TERT* mutation analysis

*BRAF* and *TERT* mutation data for the DTC cohort was available from our previous studies [20, 21].

### Statistical analysis

The associations between clinico-pathological variables and BIR was performed using contingency table analysis and Chi square tests or Mann-Whitney U test for categorical and continuous variables, respectively. Disease-free survival (DFS) was determined using Kaplan-Meier estimates. DFS was defined as the time from diagnosis to the occurrence of recurrent disease or death. Logistic regression analysis was used for analyzing the prognostic factors that could predict BIR and structural persistent disease, in univariate and multivariate manner. Two-sided tests were used for statistical analyses with a limit of significance defined as p value < 0.05. Data analyses were performed using the JMP14.0 (SAS Institute, Inc., Cary, NC) software package.

Receiver operating characterisitcs (ROC) curve analysis was performed using MedCalc software, version 10.4.7.0 for Windows (MedCalc, Ostend, Belgium).

## Results

### Patient and tumor characteristics

Mean age of the entire cohort was 38.9 years, with a male: female ratio of 1:3. 32.7% (421/1286) of tumors were bilateral and 49.9% (642/1286) were multifocal. Extrathyroidal extension was noted in 42.9% (552/1286) of DTCs. Regional lymph node metastasis (LNM) was noted in 51.6% (664/1286) of cases and distant metastasis at diagnosis was present in 5.6% (72/1286). Frequency of *BRAF* and *TERT* mutations was 52.2% (672/1286) and 11.7% (150/1286), respectively. 49.0% (630/1286) of the DTC patients received RAI therapy within 3 months of surgery (Table 1).

### Clinico-pathological characteristics associated with BIR

BIR was noted in 20.7% (266/1286) of DTCs and was significantly associated with male gender (*p* = 0.0105), larger tumor size (*p* = 0.0003), bilateral tumors (*p* = 0.0025), multifocality (*p* = 0.0214), extrathyroidal extension (*p* = 0.0013), lymph node metastasis (*p* < 0.0001), distant metastasis at diagnosis (*p* < 0.0001), advanced tumor stage (*p* = 0.0025), higher presTg levels (*p* < 0.0001), ATA high-risk category (*p* = 0.0089) and TERT mutation (*p* < 0.0001). Interestingly, we also found a significant association between BIR and ≥ three months interval to initiation of RAI therapy after surgery (*p* < 0.0001) (Table 2).

**Table 2:** Association between clinico-pathological parameters with biochemical incomplete response

Clinico-pathological variables	Biochemical incomplete response (n = 266)	Complete response (n = 1020)	*p* value
Age at diagnosis, years (mean ± SD)	39.3 ± 15.4	37.2 ± 20.0	0.1113
Gender			
Male	83 (31.2%)	239 (23.4%)	0.0105
Female	183 (68.8%)	781 (76.6%)	
Tumor diameter (cm)	3.4 ± 2.3	2.9 ± 1.9	0.0003
Tumor laterality			
Unilateral	155 (59.2%)	703 (69.1%)	0.0025
Bilateral	107 (40.8%)	314 (30.9%)	
Tumor focality			
Unifocal	114 (43.5%)	524 (51.5%)	0.0214
Multifocal	148 (56.5%)	494 (48.5%)	
Extrathyroidal extension			
Present	136 (52.1%)	416 (41.0%)	0.0013
Absent	125 (47.9%)	599 (59.0%)	
pN			
N0	61 (27.0%)	438 (46.7%)	<0.0001
N1	165 (73.0%)	499 (53.3%)	
Distant metastasis at diagnosis			
Present	32 (12.0%)	40 (3.9%)	<0.0001
Absent	234 (88.0%)	980 (96.1%)	
Stage			
I	206 (77.4%)	881 (86.5%)	0.0025
II	39 (14.7%)	98 (9.6%)	
III	3 (1.1%)	11 (1.1%)	
IV	18 (6.8%)	29 (2.8%)	
presTg (ng/ml)	159.3 ± 237.2	8.4 ± 19.9	<0.0001
*BRAF* mutation			
Present	122 (50.8%)	550 (56.6%)	0.1091
Absent	118 (49.2%)	422 (43.4%)	
*TERT* mutation			
Present	50 (21.7%)	100 (10.8%)	<0.0001
Absent	181 (78.3%)	829 (89.2%)	
Interval to RAI therapy			
<3 months	100 (37.6%)	530 (52.0%)	<0.0001
≥3 months	166 (62.4%)	490 (48.0%)	
ATA risk stratification			
Low	27 (10.2%)	171 (16.8%)	0.0089
Intermediate	90 (33.8%)	360 (35.3%)	
High	149 (56.0%)	489 (47.9%)	

All the significant variables for BIR on univariate analysis were included for multivariate analysis, except for stage since it depends on other variables such as extrathyroidal extension, LNM and distant metastasis (which are already included for multivariate analysis). On multivariate logistic regression analysis, male gender (Odds ratio (OR) = 1.60; 95% confidence interval (CI) = 1.07–2.39; *p* = 0.0231), lymph node metastasis (LNM) (OR = 1.61; 95% CI = 1.06–2.45; *p* = 0.0267), higher presTg levels (OR = 1.05; 95% CI = 1.04–1.06; *p* < 0.0001) and interval to initiation of RAI therapy of ≥ three months (OR = 2.24; 95% CI = 1.51–3.32; *p* < 0.0001) were found to be independent predictors of BIR (Table 3).

**Table 3:** Multivariate logistic regression analysis for clinico-pathological predictors of biochemical incomplete response

Covariate	Odds ratio	95% confidence interval	*p* value
Sex			
Male (vs. Female)	1.60	1.07–2.39	0.0231
Tumor laterality			
Bilateral (vs. unilateral)	1.66	0.94–2.91	0.0791
Tumor focality			
Multifocal (vs. unifocal)	1.00	0.57–1.75	0.9954
Extrathyroidal extension			
Present (vs. absent)	1.06	0.67–1.69	0.8003
Tumor size			
(per unit change)	0.95	0.85–1.05	0.2934
Lymph node metastasis			
Present (vs. absent)	1.61	1.06–2.45	0.0267
Distant metastasis at diagnosis			
Present (vs. absent)	0.78	0.32–1.89	0.5801
presTg level			
(per unit change)	1.05	1.04–1.06	<0.0001
TERT mutation			
Present (vs. absent)	1.38	0.79–2.42	0.2607
Interval to RAI therapy			
≥ 3 months (vs. < 3 months)	2.24	1.51–3.32	<0.0001
ATA risk stratification			
Low	1.00	Reference	
Intermediate	1.12	0.69–1.83	0.6405
High	2.32	0.98–5.48	0.0554

### Diagnostic accuracy of presTg for predicting BIR

**Fig. 1:**
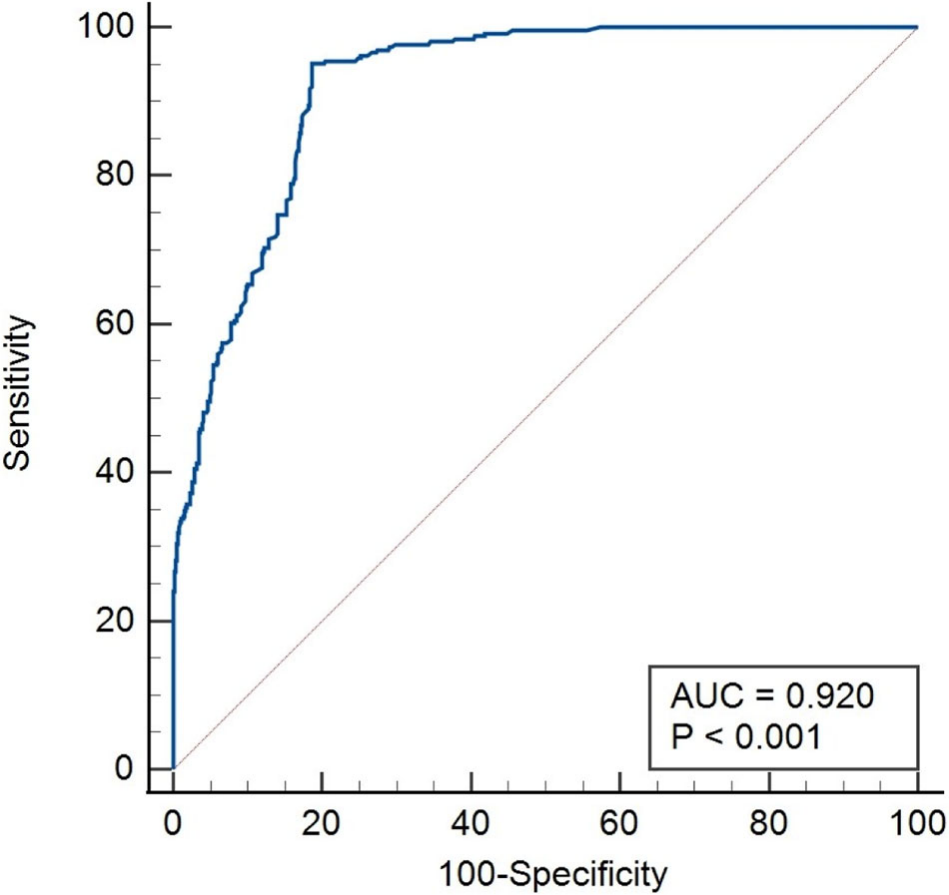
Receiver operating characteristics (ROC) curve. Pre-ablative stimulated thyroglobulin (presTg) level >10 ng/ml was shown to be an accurate predictor of biochemical incomplete response (area under the curve = 0.920, sensitivity = 95.1%, specificity = 81.3%, *p* < 0.001)

ROC curve was drawn to determine the diagnostic accuracy and cut-off value of presTg in identifying BIR in our cohort. Area under the curve (AUC) was 0.920 (95% CI = 0.904–0.934, *p* < 0.001) (Fig. 1). From the available cut-off values for presTg, the best cut-off value was ≥10 ng/ml (sensitivity = 95.1%, specificity = 81.3%). Negative predictive value (NPV) and positive predictive value (PPV) for BIR at presTg cut-off of 10 ng/ml were 98.5% and 57.0%, respectively. However, a cut-off of ≥ 6.9 ng/ml had a higher sensitivity (96.2%) and a reasonable specificity (73.8%), with NPV and PPV of 99.2% and 46.0%, respectively. The unadjusted odds ratio for BIR at presTg level of 10 ng/ml and 6.9 ng/ml was 84.5 (95% CI = 47.3–150.7, *p* < 0.0001) and 101.6 (95% CI = 44.7–230.7, *p* < 0.0001), respectively.

### Long-term clinical outcomes in patients with BIR

We next sought to determine the incidence and associations of structural disease in patients with BIR during follow-up. During a median follow-up of 9.8 years, 36.8% (98/266) of patients with BIR developed structural disease. On multivariate logistic regression analysis, male gender (OR = 1.56; 95% CI = 1.05–2.30; *p* = 0.0272) and increasing Tg levels during follow-up (OR = 4.25; 95% CI = 1.93–10.82; *p* = 0.0001) were independent predictors of structural disease in patients with BIR (Table 4).

**Table 4:** Predictors of structural disease in patients with biochemical incomplete response

Covariate	Univariate analysis		Multivariate analysis	
	OR (95% CI)	*P* value	OR (95% CI)	*P* value
Age (≥55 years)	2.10 (1.37–3.15)	0.0010	1.49 (0.90–2.39)	0.1214
Sex (male)	1.53 (1.05–2.19)	0.0254	1.56 (1.05–2.30)	0.0272
Laterality (Bilateral)	1.02 (0.70–1.48)	0.9000		
Focality (Multifocal)	0.94 (0.66–1.36)	0.7539		
Extrathyroidal extension (present)	1.15 (0.80–1.66)	0.4380		
Lymphovascular invasion (present)	1.14 (0.77–1.66)	0.5153		
LN metastasis	1.07 (0.70–1.67)	0.7493		
Tumor size (>2 cm)	1.45 (0.96–2.23)	0.0783		
Distant metastasis	1.42 (0.79–2.36)	0.2291		
TNM staging				
I	1.00 (reference)			
II	0.67 (0.23–1.55)	0.3729		
III	3.03 (0.87–5.61)	0.0958		
IV	3.00 (0.81–4.88)	0.1024		
BRAF mutation	1.19 (0.82–1.72)	0.3544		
TERT mutation	2.05 (1.33–3.07)	0.0015	1.31 (0.80–2.11)	0.2748
According to changes in serum Tg and TgAb values				
Increasing TgAb group	1.00 (reference)		1.00 (reference)	
Decreasing Tg group	1.60 (0.89–2.40)	0.0760	1.43 (0.93–2.23)	0.1066
Increasing Tg group	5.47 (3.03–10.65)	< 0.0001	4.25 (1.93–10.82)	0.0001
ATA risk stratification				
Low	1.00 (reference)		1.00 (reference)	
Intermediate	1.27 (0.67–2.59)	0.4736	0.73 (0.36–1.58)	0.4051
High	2.17 (1.23–2.59)	0.0065	1.35 (0.74–2.71)	0.3499

*OR* idds ratio, *CI* confidence interval

**Fig. 2:**
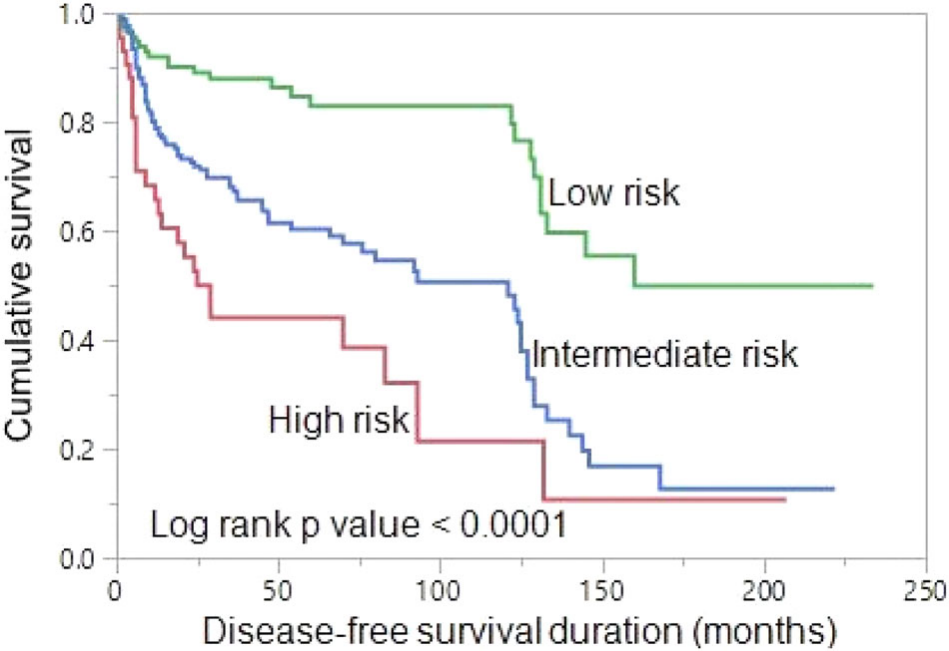
Disease-free survival (DFS). The 10-year DFS rates in the low-, intermediate-, and high-risk groups are 82.3%, 50.6%, and 21.4%, respectively. DFS is significantly better in low-risk group patients than in high-risk group patients and intermediate-risk group patients (*p* < 0.0001)

Based on the number of independent risk factors (male gender and increasing Tg levels during follow-up), patients were divided into 3 groups: low risk (no risk factors); intermediate risk (any one risk factors); and high risk (both risk factors). Risk stratification was performed for 266 patients with BIR with regards to DFS. 36.8% (98/266), 51.1% (136/266) and 12.0% (32/266) of patients were classified as low-, intermediate- and high-risk, respectively. 10-year DFS rates in the low-, intermediate- and high-risk groups were 82.3%, 50.6%, and 21.4%, respectively. DFS was significantly different among the three risk group, being worst in high-risk group (*p* < 0.0001) (Fig. 2).

## Discussion

RAI therapy is a well-established therapeutic modality for DTC patients. Despite excellent prognosis, a significant percentage of DTCs develop biochemical incomplete response (BIR) [18, 22, 23]. Data on prevalence of BIR, clinico-pathological and biochemical risk factors to predict BIR after surgery and before radioiodine ablation in DTC patients from Middle Eastern ethnicity is not fully explored. Therefore, we conducted this retrospective study to evaluate BIR prevalence as well as predictive markers after surgery and before RAI therapy. In addition, we also evaluated long-term clinical outcomes of DTC patients showing BIR following initial therapy.

In this retrospective study of 1286 DTC patients, we noted that 20.7% (266/1286) of the patients show BIR. This incidence is consistent with a previous report [24]. However, a recent study has identified higher incidence (~36%) of BIR and have attributed this to the fact that most of their patients belong to intermediate or high risk categories [18]. In our study as well, ~90% of BIR patients are in the intermediate or high-risk category.

We further evaluated multiple clinico-pathological, biochemical and molecular markers to predict the risk of BIR before radioiodine ablation. In our study, we noted four risk factors as significant predictors of BIR on multivariant analysis: presTg, the time interval between thyroidectomy and first dose of RAI therapy (≥ 3 months), male gender, and LNM. Several previous reports have identified the impact of post-operative TSH stimulated Tg in predicting persistent and/or recurrent disease in DTC [25–28]. Our result is consistent with a recent study that found BIR risk to be very high in patients who had high presTg [18].

In our study, we found that presTg ≥6.9 ng/ml had 96.2% sensitivity to predict BIR prior to RAI ablation. We further noted that low presTg (<6.9 ng/ml) had excellent NPV (99.2%) to rule out BIR. We found reasonable specificity for presTg cutoff ≥10 ng/dl (specificity = 80.6%). As opposed to high NPV, the PPV of presTg over 10 ng/ml was quite low (57%). However, the main value of presTg is as a negative predictor of BIR when presTg values are low. Although the predictive value of presTg below 10 ng/ml (which is routinely used) has been demonstrated in our analysis, it is likely that lower cutoffs such as 6.9 ng/ml suggested by ROC curve would demonstrate an even higher NPV, albeit for a smaller group of patients.

Interestingly, our study has identified that the timing of initiating radioiodine adjuvant therapy could be a significant predictor of BIR in patients from this ethnicity. Although, the current DTC guidelines have no recommendation for timing of RAI [7], several previous studies have explored the relationship between RAI initiating time and DTC clinical outcome with conflicting conclusions [23, 29–31]. Our study showed that delayed initial RAI (≥ 3 months after thyroidectomy) was an independent predictor of BIR in this cohort.

Another interesting finding in this study was the association between BIR and male gender. Sex disparity in the incidence of DTC and its effect have been well documented [32–34]. However, the impact of gender on DTC from Middle Eastern ethnicity appears to be more pronounced as we have identified in our recent study [35] that male sex was an independent prognostic factor for recurrence-free survival in PTC.

We found that presence of LNM was an independent predictor of BIR in this cohort. This is in concordance with several previous studies, where LNM was shown to be associated with unfavorable outcome in DTC patients [36–38].

We further sought to evaluate the long-term outcome of BIR patients in this cohort. With a median follow-up of 9.8 years, 36% of patients had structural disease, which was found to be significantly associated with male gender and increasing Tg after initial therapy even in multivariant analysis. According to the number of risk factors, risk stratification related to poor DFS was attempted: low-risk group (having no risk factor), intermediate-risk-group (having any one risk factor), and high-risk group (having both risk factors). 10-year DFS rates in the low-, intermediate- and high-risk groups were 82.3%, 50.6%, and 21.4%, respectively. DFS was significantly different among the three risk groups, being worst in the high-risk group. Our findings suggest that a risk adaptive management in patients with BIR could be beneficial.

Our research has a few limitations. First, it was a single-center, retrospective study due to which bias cannot be excluded. Second, the study involved a specific ethnicity, which could prevent generalizing the findings on other patient populations.

## Conclusion

We have identified easy and robust markers to predict BIR. We found that high presTg level, LNM, delayed RAI therapy (≥ 3 months) and male gender warrant higher future probabity of BIR even before RAI therapy. In addition, DTC patients exhibiting BIR had a high risk of structure disease and hence risk adaptive management is encouraged for BIR patients. Thorough disease surveillance is required for BIR patients and close attention should be given to male patients and patients with increasing Tg since they significantly impact clinical outcome in BIR patients.
